# Spinal Deformity in Ehlers–Danlos Syndrome: Focus on Musculocontractural Type

**DOI:** 10.3390/genes14061173

**Published:** 2023-05-27

**Authors:** Masashi Uehara, Jun Takahashi, Tomoki Kosho

**Affiliations:** 1 Department of Orthopaedic Surgery, Shinshu University School of Medicine, Matsumoto 390-8621, Nagano, Japan; jtaka@shinshu-u.ac.jp; 2 Department of Medical Genetics, Shinshu University School of Medicine, Matsumoto 390-8621, Nagano, Japan; ktomoki@shinshu-u.ac.jp; 3 Center for Medical Genetics, Shinshu University Hospital, Matsumoto 390-8621, Nagano, Japan; 4 Division of Clinical Sequencing, Shinshu University School of Medicine, Matsumoto 390-8621, Nagano, Japan; 5 Division of Instrumental Analysis, Research Center for Advanced Science and Technology, Shinshu University, Matsumoto 390-8621, Nagano, Japan

**Keywords:** spinal deformity, Ehlers–Danlos syndrome, musculocontractural

## Abstract

Spinal deformity in Ehlers–Danlos syndrome (EDS) is an important symptom that can lead to trunk balance deterioration, respiratory dysfunction, and digestive disorders as the deformity progresses, thereby reducing a patient’s quality of life and activities of daily living. The severity of the deformity varies widely, with treatment depending on the extent and the presence of associated complications. The present review addressed the current state of clinical research and treatment of spinal deformities in EDS with a specific focus on the musculocontractural type. Further studies are needed to better understand the underlying mechanisms of spinal deformity in EDS.

## 1. Introduction

Ehlers–Danlos Syndrome (EDS) is an inherited connective tissue disease characterized by hyperextensibility of the skin and joints and fragility of various tissues. EDS was classified into 14 disease types based on clinical presentation and molecular genetic background in the most recent 2017 classification [1,2]. Spinal deformity is a common feature in several types of EDS. The condition is typically progressive and can be severe, often leading to a deterioration of body trunk balance. As a result, many patients suffer significant reductions in quality of life (QOL) and activities of daily living (ADL). 

A 20-year follow-up case report of spinal deformity in EDS described that missed opportunities for surgical treatment of progressive spinal deformity could result in chronic deformity advancement and life-threatening respiratory failure [3]. The pathophysiology of spinal deformity in EDS is primarily due to muscle hypotonia and ligament laxity, which can lead to scoliosis ranging from mild to severe curvatures requiring operative treatment [4]. 

The finding that sagittal spinal deformity in the elderly is strongly correlated with health-related QOL [5,6] has led to the worldwide practice of corrective fusion surgery using spinal instrumentation. The Scoliosis Research Society-Schwab classification published in 2012 provides spine surgeons with an index of spinopelvic parameters to better define spinal sagittal alignment and correction goals [7]. Since its release, the number of corrective fusion procedures for spinal deformity has increased with the advancement of spinal implants, thereby improving the ADL of afflicted patients. However, corrective fusion is very invasive, and its complication rate is approximately 25% in a survey of surgical complications in adult spinal deformity by the Japanese Society for Scoliosis Research [8]. Indeed, complications in adult spinal deformity correction are not rare. In patients having EDS with spinal deformity, in addition to the risks inherent in adult spinal deformity, there can also be unique complications associated with the tissue fragility characteristic of EDS. 

Previous reports on spinal deformities in EDS have mostly been case reports or series and lack extensive scientific evidence. We particularly focused on spinal deformity in musculocontractural EDS (mcEDS) in this review since there have been no other studies on this condition apart from our case series and because the spinal deformity is more advanced than in other forms of EDS, which can severely impair patient ADL. In this article, we review the spinal deformities associated with EDS patients with a particular emphasis on clinical perspectives to gain a deeper understanding of the disorder. 

### 1.1. The General Information of EDS Types

Beighton et al.’s thorough classification of EDS has revealed a wide variety among six primary forms of the condition, each with a distinctive set of symptoms and traits [9]. The classic form of EDS is an inherited connective tissue disorder that is autosomal dominantly passed to offspring. EDS type I refers to the gravis version of the illness, which is frequently accompanied by more severe symptoms. EDS type II, on the other hand, refers to the less severe form of mitis typically manifesting with milder symptoms [9].

The type III hypermobility form of EDS is distinguished by excessive joint mobility, persistent joint discomfort, and other musculoskeletal problems. Meanwhile, a higher risk of life-threatening consequences, such as artery and organ rupture, is associated with the vascular variant of EDS known as type IV [9].

Beighton also identified a number of unique EDS varieties in addition to the primary ones. These include the type VI form of kyphoscoliosis, often known as kyphoscoliotic EDS, which is characterized by significant spinal curvature and joint deformities. Both the VIIA and VIIB form of arthrochalasia typically exhibit extreme joint instability and dislocation. Lastly, the dermatosparaxis form of EDS is categorized as type VIIC and is distinguished by anomalies of the connective tissues along with exceedingly brittle skin [9].

Other variations of the illness have been discovered in addition to the aforementioned types of EDS. These include X-linked EDS (type V), which is inherited through the X chromosome and often has a more severe impact on men. Severe gum disease and tooth loss are characteristics of the periodontitis type of EDS (type VIII). A lack of fibronectin causes the fibronectin-deficient type of EDS (type X), resulting in defects related to connective tissue form and function. Progeroid EDS and an undetermined form are other kinds of EDS that have been uncovered to date. Family hypermobility syndrome (EDS type XI) is a very recently recognized form of EDS that is characterized by joint hypermobility and persistent discomfort [9]. 

### 1.2. Frequency and Characteristics of Spinal Deformity in EDS

Idiopathic scoliosis affects between 0.47% and 5.2% of children aged 8 to 15, according to various studies [10,11,12]. One meta-analysis conducted in 2010 revealed a mean prevalence of adolescent idiopathic scoliosis of 5% in the general population [13]. 

The pathological types of EDS in which spinal deformities are relatively common are presented in Table 1. The EDS forms have been categorized and recategorized in light of the addition of new subtypes following the discovery of gene mutations. The International Consortium released a novel classification system for EDS in 2017, breaking it down into 13 subcategories to account for the mutations that have been discovered in genes encoding both collagen and collagen modifiers following recent advancements in molecular identification [1]. 

The inheritance pattern and causative genes for each EDS type in which spinal deformities are more frequent include the autosomal dominant inheritance hypermobile (unknown) and arthrochalasia (*COL1A1*, *COL1A2*) types, the autosomal recessive inheritance kyphoscoliotic (*PLOD1*, *FKBP14*) and brittle cornea syndrome (*ZNF469*, *PRDM5*) types, and the spondylodysplastic (*B4GALT7*, *B4GALT6*, *SLC39A13*) and musculocontractural (*CHST14*, *DSE*) types. The myopathic EDS types can be either autosomal dominant or autosomal recessive (*COL12A1*). The majority of individuals with one of the 12 different forms of EDS harbor genetic abnormalities that impact the manufacture and/or structure of fibrillar collagens, the complement cascade, and other related processes [1,14]. While structural flaws in genes encoding collagen-modifying enzymes are typically linked with autosomal recessive inheritance, structural deficiencies in genes encoding collagen-encoding enzymes and complement deficits are typically associated with autosomal dominant illnesses [15]. The diagnosis of EDS is now based on a combination of clinical assessment and phenotype-guided genetic testing, except for hypermobile EDS [16].

The frequency of spinal deformity in EDS varies depending on the EDS type. In an investigation of 58 patients with type I, II, III, or IV EDS corresponding to classical (typical), classical (mild), hypermobile, and vascular type, respectively, 30 (52%) patients displayed clinical and radiographic evidence of scoliosis, with type I having the highest percentage of patients (54%), followed next by type IV (33%), type III (30%), and type II (11%) [17]. The prevalence of spinal deformity in various types of EDS is reportedly higher than in the general population [10,11,12,13].

Progressive and severe spinal deformity frequently occurs in kyphoscoliotic EDS, typically presenting at birth or within the first year of life [18]. In contrast, mild and soft scoliosis is found in roughly half of the patients with hypermobile EDS; the condition progresses until puberty but rarely requires therapeutic intervention [19]. In the arthrochalasia type of EDS, deformities such as scoliosis, thoracic lordosis, and kyphoscoliosis have all been reported [14]. In brittle cornea syndrome, kyphoscoliosis is present in roughly 40% of cases [14]. In spondylodysplastic EDS, one in seven (14%) patients with a B4GALT7 deficiency had scoliosis, and 32 in 47 (68%) patients with a B3GALT6 deficiency exhibited congenital scoliosis or kyphoscoliosis occurring within the first year of life [14]. In myopathic EDS, congenital kyphosis was observed in all three patients reported, with progressive scoliosis in several cases [14]. 

A thoracolumbar sacral orthosis or brace, which is a specialized medical device designed to provide support and stability to the spine, is typically used to support coronal curves between 25° and 45° to stabilize the spine and prevent worsening [20]. The orthosis helps prevent curvature progression, thereby improving patient QOL. Numerous studies have demonstrated the efficacy of such devices in treating adolescent idiopathic scoliosis by contributing to a significant reduction in curve deterioration and enhancement of overall well-being [21,22]. 

On the other hand, the effectiveness of bracing therapy in syndromic scoliosis may be limited because the deformity occurs earlier in life and is often more progressive than in idiopathic scoliosis. One study showed that bracing was most effective in children with a spinal curvature above 20°–25° who were too old for casting in a cohort of scoliosis patients with Prader–Willi syndrome [23]. Bracing is intended to stop curve advancement. However, in cases of very large curves, braces may be used to maintain the curve until the patient is ready for surgery [23]. Generally, 5 of less than 30° are treated with a Providence-style brace that is only worn at night [23]. For daytime use, a typical thoracic-lumbar-sacral orthosis is prescribed if the curvature is more than 30° [23]. If a curve advances to over 50° and a future operation is expected, brace treatment is continued as long as in-brace radiographs demonstrate a curve of under 50°, thereby giving the child a chance to grow even further [23]. Foy et al. described a female kyphoscoliotic EDS patient who was followed carefully to control scoliosis progression and for the adjustment of bracing and physiotherapy [24]. However, at 14 years of age, she received spinal fusion surgery to correct her rapidly progressing scoliosis [24]. In a case series of scoliosis patients with Loeys–Dietz syndrome reported by LoPresti, the median follow-up period was 51 months, and the median age of scoliosis diagnosis was 11.5 years [25]. The successful observational management of two patients resulted in average initial and final major coronal curves of 14° and 20.5°, respectively [25]. Both were braced, one successfully (initial major coronal curve: 15°, final major coronal curve: 30°) and the other unsuccessfully (initial major coronal curve: 40°, final major coronal curve: 58°) due to a progressive illness that required surgery [25]. One of the patients who received surgical correction underwent surgery for a spinal abnormality that was permanently corrected (major coronal curve: 33° to 19°) [25]. In another study of scoliosis patients with Loeys–Dietz syndrome, only 27% of patients had successful bracing, and more than 60% of those cases advanced to surgical curve magnitudes, with 47% having undergone surgery at the final follow-up [26]. Since no reports of successful orthotic therapy for spinal deformity in patients with Ehlers–Danlos syndrome were found during our search, we were unable to confirm the effectiveness of orthotic therapy for this disorder.

Surgery is often required for EDS patients to manage respiratory difficulties brought on by advancing kyphoscoliosis [27]. To prevent intraoperative vascular damage that can cause spinal cord ischemia or postoperative neurological impairments, surgeons should be mindful of vascular problems in addition to generalized tissue fragility and bruisability. 

Surgically correcting kyphoscoliosis carries a significant risk of paraplegia and catastrophic artery rupture in patients with EDS [27,28,29]. Akpinar et al. described two serious vascular complications among five patients with EDS type VI (kyphoscoliotic type) who underwent surgical treatment for spinal deformity [27]. In one case, avulsion of the segmental artery occurred, and the iliac artery and vein ruptured during anterior surgery [27]. In the other case, avulsion of the superior gluteal artery was encountered during subperiosteal dissection to harvest iliac bone [27]. Vogel et al. reported on three neurological complications and one vascular complication that occurred during spinal deformity surgery of EDS type VI patients [28]. Two of the three (67%) cases with neurologic complications suffered permanent paraplegia [28]. In the patient with vascular complications, two segmental arteries were dissected from the inferior aorta during anterior surgery [28]. In a study by Yang et al. on surgical cases of spinal deformity in EDS, all three patients displayed massive bleeding, with blood loss ranging from 600 mL to 6 L [29]. Since all of the above complications occurred without any apparent cause, such as intraoperative procedural error, it was considered that neurovascular injuries could occur as a specific event in EDS despite precautionary measures.

Hypotonia of the muscles and lax ligaments were thought to be the main contributors to spinal deformity in a prior series of kyphoscoliotic EDS [30]. On the other hand, vertebral fractures were noted in 39% of individuals in a large series that predominantly included patients with hypermobile and classical EDS, which was a significantly higher frequency when compared with control subjects (38.5% vs. 5.1%; *p* = 0.001) [31]. Chondroitin sulfate N-acetylgalactosaminyltransferase-1 (T1) is a key glycosyltransferase in chondroitin sulfate biosynthesis [32,33]. In a study of T1 knockout mice exhibiting such EDS-like connective tissue defects as hyperstretching of the skin, all test mice had severe scoliosis [34]. Another study using mouse models of EDS showed that the *Nell 1* gene caused narrowing of the disc space and spinal curve alterations in *Nell 1*-deficient mice [35]. Other genes expressed during fetal life that are affected by *Nell 1*, including *Tnrsf11b* and *Bmpr1a*, are also known to be important in human and mouse vertebral body development [36,37]. However, the mechanisms of spinal deformity in EDS remain far from establishment.

## 2. Materials and Methods

Henceforth, we will first provide a general introduction to mcEDS. Afterward, we will present the clinical results of three surgical cases of spinal deformity in mcEDS. We will then discuss the characteristics of spinal deformity surgery in mcEDS and highlight several important considerations in perioperative management.

### 2.1. Musculocontractural EDS

In the 2017 international classification of EDS with 13 different variants, mcEDS was introduced as a new EDS type distinguished by progressive connective tissue fragility, including skin hyperextension and fragility, generalized joint laxity, joint dislocation, joint deformity, and massive subcutaneous hematoma, in addition to such developmental abnormalities as facial features, congenital multiple joint contractures, and visceral and audiovisual congenital complications [38,39]. Miyake et al. identified the causative gene of mcEDS as *CHST14*, encoding dermatan 4-*O*-sulfotransferase 1 (D4ST1) [40]. D4ST1 is an enzyme that adds a sulfate group to the 4-position of the N-acetylgalactosamine residue of dermatan sulfate, which is a component of glycosaminoglycan side chains in proteoglycans [41,42]. Miyake et al. revealed progressive connective tissue fragility to be caused by a loss of function mutation in *CHST14*, which resulted in defective assembly of collagen fibers via decorin through an altered composition of the glycosaminoglycan chains attached to decorin, as well as mcEDS being caused by a dermatan sulfate deficiency [40]. Sixty-six patients from 48 families with mcEDS have been reported worldwide to date, indicating the global significance and medical relevance of these findings [43]. 

### 2.2. Spinal Deformity in mcEDS

**Figure 1:**
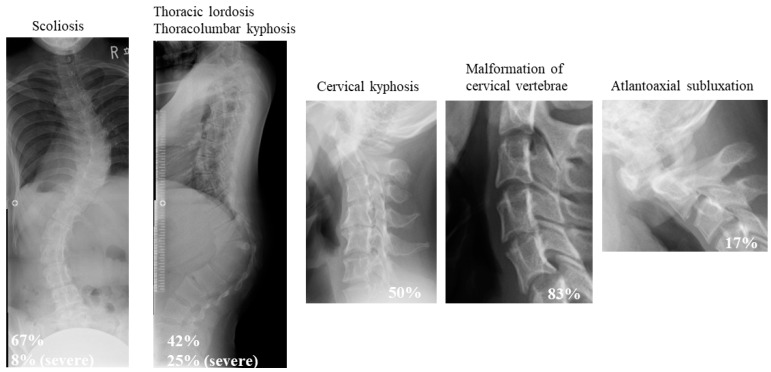
Features of spinal disorders in patients with mcEDS. Adapted from [42], with permission from Wiley, 2023.

Early-onset kyphoscoliosis, a condition commonly found in mcEDS patients, often results in severe deformity, trunk balance deterioration, respiratory dysfunction, and digestive disorders. Thus, it is one of the most prominent symptoms reducing patient QOL and ADL and requires appropriate corrective treatment. Since the disease is characterized by progressive connective tissue fragility, there is concern that massive bleeding from hemorrhage and bone fragility may lead to implant dislocation and pseudoarthrosis during treatment. In a study summarizing the extent and characteristics of spinal deformity in 12 patients with mcEDS, cervical kyphosis (50%), cervical vertebral body deformity (83%), atlantoaxial subluxation (17%), advanced kyphosis at the thoracolumbar transition (42%), and scoliosis (67%) were found to be the most characteristic (Figure 1) [44]. Three (25%) patients had severe thoracolumbar kyphosis with a kyphotic angle of more than 50° along with thoracic lordosis and a wedge-shaped spinal deformity at the thoracolumbar junction; two of them underwent surgical correction of the deformity [44]. Bone tissue observation and bone morphometry revealed no calcification disorders and reduced bone metabolic turnover [38]. In another report of three surgical cases of severe spinal deformity in mcEDS, a tendency for massive subcutaneous hemorrhage and intraoperative bleeding due to fragile skin were problems specific to this condition [45]. Careful attention to positional changes and intraoperative hemorrhage control was necessary to address these problems [45]. Surgical planning was carried out considering a two-stage operation, with a total blood loss volume of 2 L. Additionally, 1-desamino-8-D-arginine vasopressin was also administered to prevent subcutaneous hematoma [45]. 

The paravertebral muscles are essential for spinal stability and functional movement, and imbalances in their tension are considered to contribute to idiopathic scoliosis [46]. In age-related adult spinal deformity, whole-body muscle mass and paravertebral muscle area and fatty degeneration on magnetic resonance imaging have been implicated in deformity progression [47,48]. In recent years, there have been significant advancements in the creation of mouse models aimed at studying this particular disease [49,50,51]. Despite the progress made, however, the precise pathogenic mechanisms are yet to be fully understood and require further investigation at the molecular level. Novel mouse models represent a valuable resource in future research to unravel the complexities of spinal deformity development in mcEDS.

## 3. Results

### Surgical Case Presentations of Spinal Deformity in mcEDS

Severe spinal deformities in patients with mcEDS often significantly diminish QOL and ADL, thus necessitating appropriate corrective surgery. We present three surgical cases of severe spinal deformity in mcEDS that were described in our earlier publications [44,45]. 

**Figure 2:**
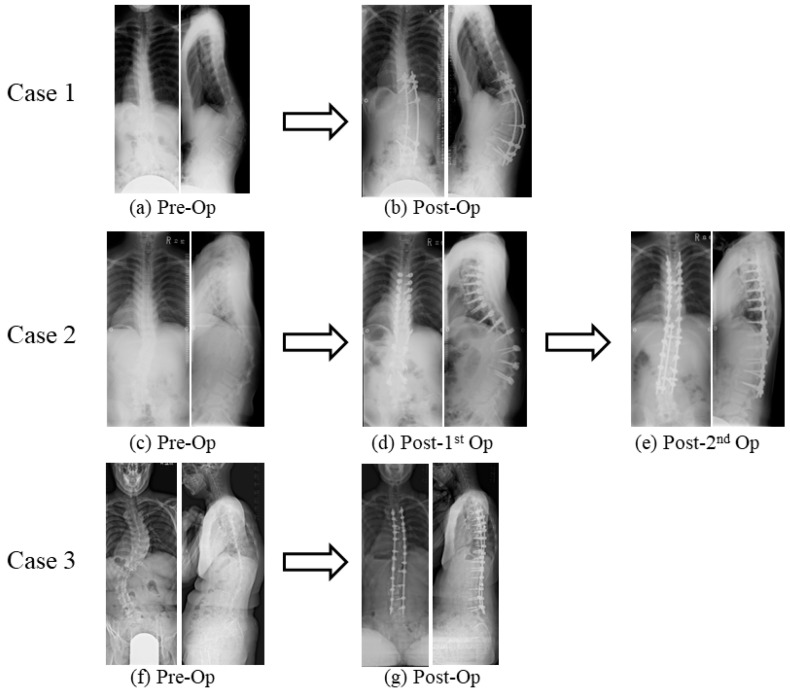
Surgical cases of spinal deformity in mcEDS. (**a**,**b**) Case 1: Posterior spinal corrective fusion was performed using pedicle screws from T10 to L4. (**c**–**e**) Case 2: Two-stage posterior spinal corrective fusion was carried out using pedicle screws from T4 to L3. (**f**,**g**) Case 3: Posterior spinal corrective fusion was performed using pedicle screws from T4 to L4. Reproduced from [42,43], with permission from Wiley and Elsevier, 2023.

Case 1: At the age of 16 years, a female patient presented with scoliosis and thoracolumbar kyphosis, which caused her to experience severe back pain. Upon examination, we found that she had a major coronal curve of 33° and a kyphotic angle of 57° (Figure 2a) [44]. The Preoperative Scoliosis Research Society-22 (SRS-22) domain evaluation provided a comprehensive assessment of the patient’s postoperative condition, encompassing multiple aspects such as physical function, pain, self-image, and mental well-being. Her function score was 2.4 (maximum value: 5.0), pain score was 3.8, self-image score was 1.6, mental health score was 2.6, and subtotal score was 2.6. In order to correct her spinal deformity, we performed posterior spinal corrective fusion using pedicle screws from T10 to L4. The surgery lasted 266 min and resulted in a blood loss of 1550 g [44]. Following the procedure, we observed a significant improvement in the patient’s condition, with the major coronal curve and kyphotic angles being reduced to 8° (correction rate: 76%) and 46° (correction rate: 19%), respectively (Figure 2b) [44]. During the surgical procedure, we employed intraoperative neuromonitoring techniques but refrained from administering antifibrinolytic medications. In the perioperative period, the patient received a total of six units of red blood cells (RBC) and two units of fresh frozen plasma (FFP) without any apparent neurovascular complications. However, during the process of moving the patient to a prone position, an arterial line was removed, resulting in a massive subcutaneous hematoma. Following the surgery, it was discovered that a screw had caused a fistula, which subsequently became infected at the apex of the kyphosis and necessitated surgical removal. Even after 8.8 years, the fistula remained and required oral antibiotics. Despite the patient reporting a relatively low level of back discomfort as measured by a visual analogue scale (VAS) score of 65/100, the thoracolumbar kyphosis had worsened, as evidenced by a kyphotic angle of 3° [44]. In the postoperative SRS-22 domain evaluation, her function score was 2.8, pain score was 4.2, self-image score was 3.4, mental health score was 3.2, subtotal score was 3.4, satisfaction score was 3, and total score was 3.4.

Case 2: At the age of 12 years, a male patient presented with scoliosis, thoracic lordosis, and thoracolumbar kyphosis, displaying a major coronal curve of 21° and a kyphotic angle of 39° [44]. Over the course of the next five years, both of these angles continued to increase, reaching 30° and 67°, respectively (Figure 2c) [44]. The patient reported severe back pain, fatigue, and difficulty walking long distances [44]. In the preoperative SRS-22 domain evaluation, his function score was 4, pain score was 5, self-image score was 3.4, mental health score was 4.4, and subtotal score was 4.2. In order to address these issues, two-stage posterior spinal corrective fusion was performed utilizing pedicle screws spanning from T4 to L3 (Figure 2d,e) [44]. To prevent the development of large subcutaneous hematomas, intranasal 1-deamino-8-D-arginine vasopressin was administered preoperatively. During the first surgery of 294 min, there was a total blood loss of 2100 g [44]. Three weeks later, the spinal correction surgery was performed, resulting in an improvement of both the major coronal curve and kyphotic angles to 14° with correction rates of 53% and 79%, respectively [44]. This second surgery lasted 261 min and caused a blood loss of 1050 g [44]. During the surgical procedure, we employed intraoperative neuromonitoring techniques to ensure the safety of the patient’s nervous system. No antifibrinolytic medications were given. In the perioperative period, he received a total of eight units of RBC, four units of FFP, and six units of autologous blood transfusion. We recorded no neurovascular complications or other adverse events. Following the surgery, the patient described a notable improvement in back pain, with a VAS score of 0 out of 100. In the postoperative SRS-22 domain evaluation, his function score was 4, pain score was 3.6, self-image score was 4.4, mental health score was 3.8, subtotal score was 4, satisfaction score was 4, and total score was 4.1. As a result, he was able to walk with greater ease. It was noteworthy that this positive outcome persisted for 2.6 years after the surgical procedure [44]. 

Case 3: A 19-year-old female patient presented with scoliosis and thoracolumbar kyphosis. The major coronal curve was measured at 69°, and the kyphotic angle was 27° (Figure 2f) [45]. When she was 14 years old, she was diagnosed as having scoliosis and received an orthopedic brace but complained of severe back pain (no scale data) and difficulty walking [45]. She could walk only 50 m with Lofstrand crutches. To prevent the occurrence of large subcutaneous hematomas, intranasal 1-deamino-8-D-arginine vasopressin was administered preoperatively. Posterior spinal corrective fusion was carried out using pedicle screws from T4 to L4. As a result, the major coronal curve and kyphotic angles improved to 26° (correction rate: 69%) and 6° (correction rate: 78%), respectively (Figure 2g) [45]. The surgery lasted for 386 min, with a blood loss of 2600 g [45]. During the procedure, we utilized intraoperative neuromonitoring to ensure optimal nerve function without antifibrinolytic medications. The patient received a transfusion of 10 units of RBC and 10 units of FFP during the perioperative period. No complications were encountered, including neurovascular events. Afterward, the patient’s back pain showed significant improvement (VAS score of 0 out of 100) [45]. In the postoperative SRS-22 domain evaluation, her function score was 4.0, pain score was 5.0, self-image score was 4.4, mental health score was 4.4, subtotal score was 4.45, satisfaction score was 4.5, and total score was 4.45. One year post-surgery, the correction remained in place, and the patient was able to walk for approximately 30 min without difficulty [45].

## 4. Discussion

Based on the above cases, posterior spinal corrective fusion appears to be a viable treatment option for severe spinal deformity in patients with mcEDS. All patients showed improvements in low back pain after surgery (VAS score of 65 in Case 1, 10 in Case 2, and 0 in Case 3), although the gains in Case 1 were relatively modest. Due to connective tissue fragility, extreme caution must be exercised to avoid massive blood loss. The mean total intraoperative blood loss was 2433 g in our mcEDS cases, which was higher than the reported blood loss volume in correction surgery for patients with idiopathic scoliosis (1060 ± 688 g) [52]. The mean blood loss volume in other case studies of scoliosis in EDS patients has ranged from 1243 g to 1764 g [25,53,54]. Surgeons should perform careful hemostasis, and two-stage surgery may be required if bleeding is substantial. Early blood transfusions should be administered as necessary. Although reports of spinal deformity surgery in mcEDS patients are few, the deformity often becomes severe and surgical invasiveness tends to be high. Further research is warranted to establish safe surgical management protocols for spinal deformity surgery in mcEDS.

## 5. Conclusions

This review provides a comprehensive summary of the current state of clinical research on spinal deformities in individuals with EDS, especially with mcEDS. EDS-associated spinal alterations can be progressive and severe, and complications associated with tissue fragility should be carefully considered during surgery. Surgical treatment for EDS and related spinal deformities is very challenging, with risks such as massive bleeding, but it is an essential step towards maintaining patient ADL. Particular attention is needed for the potential of neurovascular injury from the stretching of fragile soft tissues, which are a hallmark of EDS. Despite significant progress in our understanding of the mechanisms that underlie the progression of spinal deformities in EDS, further research is needed to improve our knowledge and clinical decision-making.

## Figures and Tables

**Table 1:** Types of EDS in which spinal deformities are relatively common.

Clinical Type	Genetic Pattern	Causative Gene(s)
Kyphoscoliotic (Type VI)	AR	*PLOD1* *FKBP14*
Hypermobile (Type III)	AD	Unknown
Arthrochalasia (Type VIIA, VIIB)	AD	*COL1A1* *COL1A2*
Brittle cornea syndrome	AR	*ZNF469* *PRDM5*
Spondylodysplastic	AR	*B4GALT7* *B4GALT6* *SLC39A13*
Myopathic	AD, AR	*COL12A1*
Musculocontractural	AR	*CHST14* *DSE*

AD, autosomal dominant; AR, autosomal recessive.

## Data Availability

Not applicable.
